# Solving neurodegeneration: common mechanisms and strategies for new treatments

**DOI:** 10.1186/s13024-022-00524-0

**Published:** 2022-03-21

**Authors:** Lauren K. Wareham, Shane A. Liddelow, Sally Temple, Larry I. Benowitz, Adriana Di Polo, Cheryl Wellington, Jeffrey L. Goldberg, Zhigang He, Xin Duan, Guojun Bu, Albert A. Davis, Karthik Shekhar, Anna La Torre, David C. Chan, M. Valeria Canto-Soler, John G. Flanagan, Preeti Subramanian, Sharyn Rossi, Thomas Brunner, Diane E. Bovenkamp, David J. Calkins

**Affiliations:** 1 Department of Ophthalmology and Visual Sciences, Vanderbilt Eye Institute, Vanderbilt University Medical Center, Nashville, TN USA; 2 Neuroscience Institute, NYU Grossman School of Medicine, New York, NY USA; 3 Neural Stem Cell Institute, NY 12144 Rensselaer, USA; 4 Department of Neurosurgery and F.M. Kirby Neurobiology Center, Boston Children’s Hospital, Harvard Medical School, Boston, MA USA; 5 Department of Neuroscience, University of Montreal, Montreal, QC Canada; 6 Department of Pathology and Laboratory Medicine, University of British Columbia, Vancouver, BC Canada; 7 Spencer Center for Vision Research, Byers Eye Institute, Stanford University, CA Palo Alto, USA; 8 F.M. Kirby Neurobiology Center, Boston Children’s Hospital, MA Boston, USA; 9 Department of Ophthalmology, University of California San Francisco, San Francisco, CA USA; 10 Department of Neuroscience, Mayo Clinic, Jacksonville, FL USA; 11 Department of Neurology, Washington University in St. Louis, St. Louis, Missouri USA; 12 Department of Chemical and Biomolecular Engineering and Helen Wills Neuroscience Institute, University of California Berkeley, Berkeley, CA USA; 13 Department of Cell Biology and Human Anatomy, University of California Davis, Davis, CA USA; 14 Division of Biology and Biological Engineering, California Institute of Technology, CA 91125 Pasadena, USA; 15 Herbert Wertheim School of Optometry and Vision Science, University of California Berkeley, Berkeley, CA USA; 16 CellSight Ocular Stem Cell and Regeneration Research Program, Department of Ophthalmology, Sue Anschutz-Rodgers Eye Center, University of Colorado, Aurora, CO USA; 17 Glaucoma Research Foundation, San Francisco, CA USA; 18 BrightFocus Foundation, Clarksburg, MD USA

**Keywords:** Neurodegeneration, Alzheimer’s Disease, Glaucoma, Parkinson’s Disease, Huntington’s Disease, Genetics, Metabolic stress, Neuro-regeneration, Neuro-replacement, Neurovascular coupling, Biomarker, Cell-replacement, Detection, Glia, Imaging, Model Systems, Organoids

## Abstract

Across neurodegenerative diseases, common mechanisms may reveal novel therapeutic targets based on neuronal protection, repair, or regeneration, independent of etiology or site of disease pathology. To address these mechanisms and discuss emerging treatments, in April, 2021, Glaucoma Research Foundation, BrightFocus Foundation, and the Melza M. and Frank Theodore Barr Foundation collaborated to bring together key opinion leaders and experts in the field of neurodegenerative disease for a virtual meeting titled “Solving Neurodegeneration”. This “think-tank” style meeting focused on uncovering common mechanistic roots of neurodegenerative disease and promising targets for new treatments, catalyzed by the goal of finding new treatments for glaucoma, the world’s leading cause of irreversible blindness and the common interest of the three hosting foundations. Glaucoma, which causes vision loss through degeneration of the optic nerve, likely shares early cellular and molecular events with other neurodegenerative diseases of the central nervous system. Here we discuss major areas of mechanistic overlap between neurodegenerative diseases of the central nervous system: neuroinflammation, bioenergetics and metabolism, genetic contributions, and neurovascular interactions. We summarize important discussion points with emphasis on the research areas that are most innovative and promising in the treatment of neurodegeneration yet require further development. The research that is highlighted provides unique opportunities for collaboration that will lead to efforts in preventing neurodegeneration and ultimately vision loss.

## Background

A wide spectrum of neurodegenerative disorders affects the central nervous system (CNS), causing a breakdown in connectivity and communication between neurons integral to sensory, motor, and cognitive processes including vision, hearing, movement, speech and language, memory, and others. This breakdown in neuronal connection is characterized by the progressive degradation of synapses and axons that lead to eventual neuronal death. Cases of neurodegeneration and dementia worldwide are predicted to rise dramatically with the aging population, posing a significant threat to global healthcare systems [1,2]. Although neurodegenerative diseases are highly complex and can be etiologically distinct, uncovering commonalities in disease mechanisms and pathologies may yield a deeper understanding of the triggering events in neurodegeneration and generate opportunities for novel pan-neurodegenerative therapeutic avenues.

## Main text

### Etiological features of neurodegenerative disorders

#### Alzheimer’s disease and related dementias

The symptoms associated with neurodegenerative disease are largely dependent on the CNS tissue affected, which varies across diseases such as Alzheimer’s Disease (AD), Huntington’s Disease (HD), Parkinson’s Disease (PD), and Amyotrophic lateral sclerosis (ALS). Although each neurodegenerative disease is distinct in terms of etiology, severity, and rate of progression, shared molecular changes and mechanisms can be identified offering potential avenues for research across multiple diseases.

Alzheimer’s Disease represents the most common form of dementia, predominantly afflicting the aged population [3]. Over time, patients develop gradual but progressive memory loss and cognitive decline associated with the degeneration of neurons [4]. In AD, severity of symptoms is correlated with pathophysiological events caused by protein aggregations in the cerebral cortex [5–8]. These have been shown histologically as the deposition of β-amyloid (Aβ) aggregated fibrils and plaques, and neurofibrillary tangles containing hyperphosphorylated Tau protein [5]. Amyloid precursor protein (APP) can be cleaved to form varying lengths (from 38 to 43 amino-acids) of Aβ peptides [9]. Aβ monomers can bind to one another to eventually form oligomers and insoluble plaques. The deposition and accumulation of Aβ oligomers is generally accepted as central to pathogenesis of AD and the most toxic to neurons; however, other pathological events such as tau aggregation, as well as neuroinflammation also play a major role and contribute to synaptic loss and neurodegeneration [3].

While AD accounts for 60–80% of dementia cases, vascular cognitive impairment and dementia (VCID) are the second leading cause of dementia [10]. Recent mounting evidence supports an underlying vascular element in the pathophysiology of AD [11]; abnormal microvasculature in AD patients is present post-mortem in the brains of patients [11–13]. In fact, the role of cerebrovascular alterations in dementia-associated neurodegenerative diseases has been highlighted as a primary cause of cognitive impairments and as a factor that contributes directly to dementia associated with neurodegeneration [14, 15].

PD, the second most common form of neurodegenerative disorder [16], is also characterized by progressive loss of neurons. Neurodegeneration in PD leads to the impairment of basal ganglia in the brain, presenting in the clinic as difficulty with motor-movement, cognitive impairment, autonomic failure and other neuropsychiatric symptoms [17]. Similar to AD, PD symptoms also correlate with aggregates of misfolded protein, in this instance α-synuclein, leading to the subsequent formation of Lewy bodies [18]. PD falls under an umbrella of synucleinopathies which also include multiple system atrophy and dementia with Lewy bodies [19].

Among neurodegenerative disorders, ALS is the most rapid to progress to fatality; where PD and AD symptoms can begin in a prodromal period that can last many years, ALS can begin and span to death in under 2–3 years [20]. ALS manifests as widespread motor neuron abnormalities involving the brain, spinal column and peripheral neuromuscular system; speech impairment, difficulty swallowing followed by progressive paralysis of the arms and legs are common [20]. Progress in therapeutics for ALS patients is slow due to the complexity and heterogeneity of disease mechanisms. Some 15% of ALS cases are familial can be directly attributed to disease-causing alleles of genes such as *SOD1*, *TARDBP*, *FUS*, and *OPTN* [20]. Pathological mechanisms in ALS include metabolic impairment (gross mitochondrial morphological and functional changes), glutamate-induced excitotoxicity, and neuroinflammation [20]. Again, in line with other neurodegenerative diseases, ALS pathophysiology also includes protein aggregation, this time of the TAR DNA-binding protein 43 (TDP43) which can occur in sporadic and familial forms of ALS [21].

The etiologies of AD, PD, ALS, and other related dementias are highly complex. In addition to the pathophysiological changes seen post-mortem, such as deposition of insoluble protein aggregations, there are overlapping and common mechanisms of neurodegeneration that include neuroinflammatory, metabolic, neurovascular, and genetic factors.

#### Neurodegeneration of the visual system

Glaucoma is the leading cause of irreversible blindness worldwide [22]. The disease encompasses a group of optic neuropathies that lead to the progressive degeneration of retinal ganglion cells (RGCs), the output neurons of the retina, along with their axons which form the optic nerve - the sole neuronal projection to the brain’s higher vision centers. Like many other neurodegenerative diseases, glaucoma is associated with increasing age; as our population ages, it is estimated that approximately 112 million people will be affected worldwide by 2040 [23]. Besides age, elevation in intraocular pressure (IOP) is amongst other prominent risk factors for the disease which include race, severe myopia, central corneal thickness, and genetic predisposition to congenital glaucoma.

Forms of glaucoma are classified clinically according to a key anatomic feature of the anterior segment, the iridocorneal angle, which is defined by the angle formed where the iris and cornea meet. In the most prevalent form of the disease, primary open-angle glaucoma (POAG), the angle is open but there is a progressive resistance within the aqueous humor outflow pathways that gradually leads to an increase in IOP. However, not all glaucoma patients suffer from elevations in IOP; normotensive glaucoma patients never experience increases in IOP [24, 25] and conversely some patients with extremes in IOP at risk for glaucoma do not exhibit neurodegeneration [26]. IOP remains the only treatable risk factor, and although interventions in the clinic such as IOP-lowering drops or IOP-lowering surgery are available, many patients progress with neurodegeneration of the visual projection despite treatment [27]. As advances are made in research we are beginning to understand that glaucoma is characterized by the *sensitivity* of the optic projection to IOP, rather than IOP itself [27]. How this sensitivity begins or evolves throughout disease progression, or which IOP-independent mechanisms are at play remain to be determined but may hold the key to early detection and prevention in the disease.

The optic nerve head (ONH), where over 1.5 million unmyelinated RGC axons converge to exit the globe and form the optic nerve proper in humans, is a critical juncture for pathogenic neurodegenerative processes that occur in glaucoma. The vulnerability of axons at this site is by virtue of the unique structure and physiology of the ONH [28–31]. There, a complex interplay is seen between neuronal, glial, vascular, and biomechanical components that can change with age to influence sensitivity of the optic projection to any given IOP [28, 29, 32, 33]. All tissues in the human body show natural variations in stiffness, and changes in this stiffness occur naturally with aging, but can also be exacerbated as a result of inflammatory events (i.e., increased deposition of collagen and extracellular matrix components by cells, or proliferation of glia, namely astrocytes). In addition, remodeled tissue and increased stiffening act as environmental cues to further drive inflammation [34]. There appears to be an interplay between inflammation and cellular biomechanics that may be relevant in glaucoma and tissues of the ONH [34]. Changes in the retina and ONH associated with mechanosensitivity [35, 36], as well as alterations in ocular stiffness with age [37], have been independently investigated in glaucoma pathogenesis, along with extracellular matrix deposition due to inflammation. Making the connection between tissue biomechanics and inflammation as a key molecular driver of pathogenesis may uncover novel areas of therapeutic intervention in glaucoma. It is also becoming apparent, in a range of neurodegenerative diseases, that the immune and glial responses are not dependent on any one genetic mutation or predisposition for disease – making understanding of these mechanisms important for all patients.

The variety in etiology of glaucoma combined with the ineffectiveness of IOP-lowering drugs for many patients suggests multiple mechanisms of neurodegeneration. By considering glaucoma a neurodegenerative disease, research into the triggers (i.e., early molecular events) and drivers of neurodegeneration can identify novel areas of therapeutic intervention to preserve and restore vision. In addition, the optic projection is an accessible extension of the CNS that allows investigators to directly visualize CNS neurons and define mechanisms that may be leveraged for understanding other neurodegenerative diseases.

### Mechanisms of progression

**Fig. 1:**
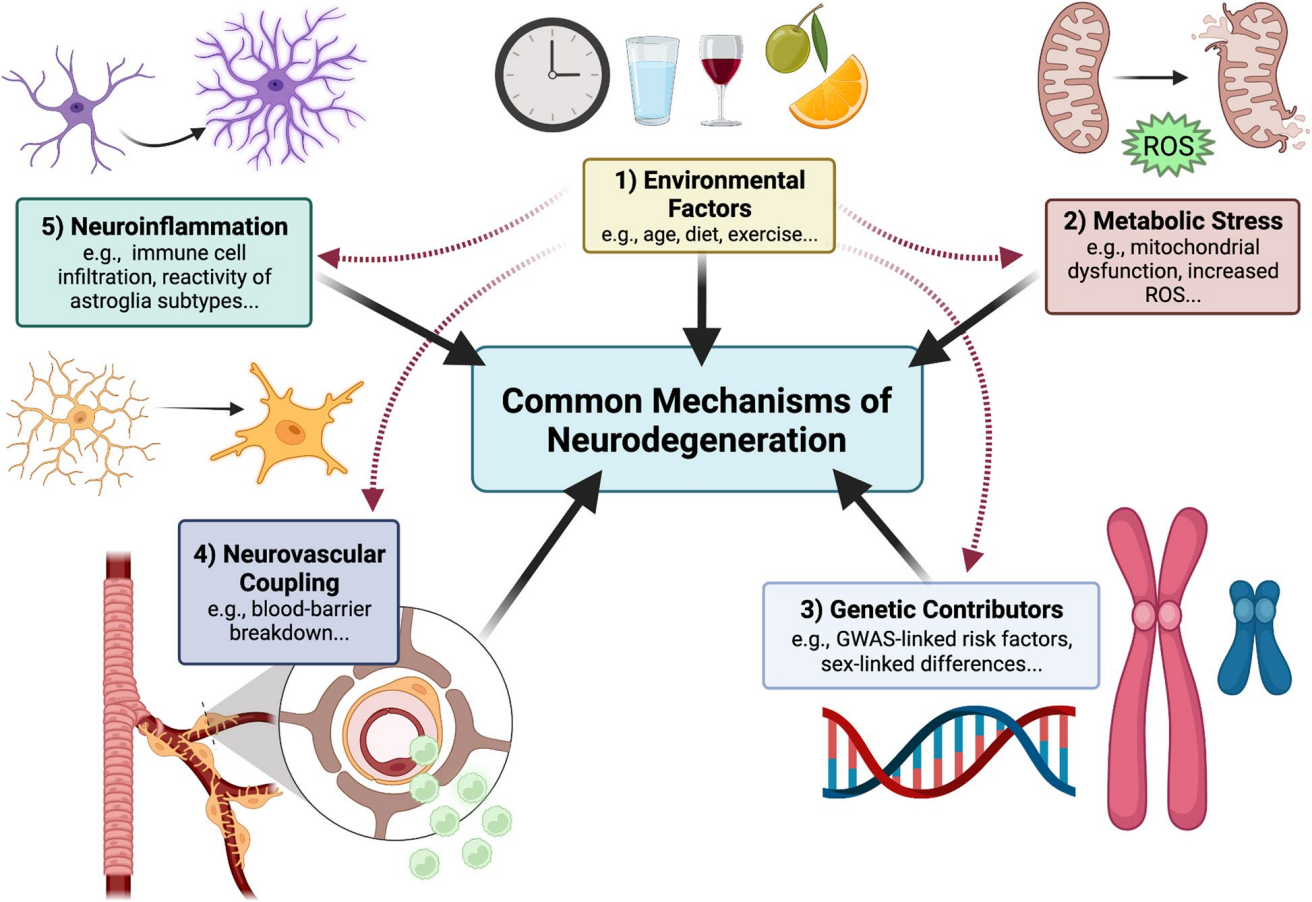
Common mechanisms of neurodegeneration. Across neurodegenerative diseases, five main areas of mechanistic overlap exist, these include: (1) environmental factors such as diet, age, and, exercise; (2) metabolic stress, e.g., mitochondrial dysfunction, increased reactive oxygen species (ROS); (3) genetic contributions, e.g., genome-wide association study-linked risk alleles (GWAS), sex-linked genetic contributions; (4) neurovascular coupling, e.g., breakdown of the blood-brain-barrier and dysfunctional neurovascular coupling and; (5) neuroinflammation, e.g., infiltration of peripheral immune cells, and increased glial reactivity. Environmental factors contribute to all mechanistic areas of degeneration

It is no coincidence that as humans age, so too does the incidence of neurodegenerative disease as homeostatic cellular mechanisms begin to malfunction, and new cellular functions associated with diseases arise. Neurodegeneration involves complex interactions between adjacent cells and their axonal projections; neurons have both proximal and distal regions that have distinct cellular environments and in turn distinct mechanisms of disease pathology. Furthermore, the CNS does not always act in isolation; the peripheral nervous system (PNS) and peripheral immune system are increasingly implicated as active players in the degeneration of the CNS. Identifying molecular commonalities will enhance understanding of neurodegenerative events, which could then be harnessed in the design of broad-stroke therapeutics for neurodegenerative mechanisms across multiple diseases. To reach this goal of broadly applicable therapeutics for neurodegenerative disease some knowledge gaps remain: (i) common molecular events in the early stages of disease progression, i.e., triggering events that tip the scale in an amplification cascade that leads to neurodegeneration, (ii) events in progression that catalyze already existing neurodegenerative events, (iii) which cell types are involved, (iv) common pathological endpoints, i.e., how can we back-track from these events to prevent or replace diseased tissue, and finally (v) discerning which events are pro-degenerative vs. reparative or even pro-regenerative. As a collective, we have identified several common mechanistic areas of focus that may provide potential pan-neurodegenerative therapeutic strategies. These include: environmental factors, neuroinflammation, metabolic stress, neurovascular coupling, and genetic contributions to disease (Fig. 1).

#### Environmental contributions to neurodegeneration

Environmental factors can have a profound impact on cellular and epigenetic contributions to disease progression. For example, these factors include age, diet, exercise, and exposure to neurotoxic substances that can act to trigger and/or exacerbate underlying neurodegenerative events. As such, environmental factors play a role in many of the shared degenerative mechanisms discussed below. Across many diseases, age is a primary risk factor and tissues that are comprised of postmitotic cells, such as neurons in the brain and retina, are particularly sensitive to the effects of aging [38]. Hallmarks of aging cells include genomic instability, epigenetic alterations, dysregulated signaling pathways, and mitochondrial dysfunction. Changes that occur with age can impact homeostatic functions in cells, rendering them sensitive to neurodegeneration. Other external factors, such as diet and exercise, are proving to be crucial factors in maintaining CNS health [39, 40]. Micronutrients, such as vitamins and trace elements are integral to many key biological processes, such as mitochondrial ATP production and immune responses, which in turn affect CNS physiology [39]. Recognizing the role that external factors play in degeneration and the impact on cellular mechanisms as outlined blow (i.e., signaling pathways such as neuroinflammation, metabolism, mitochondrial dysfunction), will help to provide novel therapeutic strategies for neurodegenerative diseases.

#### Neuroinflammation

Inflammatory events that influence the CNS (what is sometimes referred to as “neuroinflammation”) have multifaceted outcomes, which can be neuro-protective, neuro-regenerative and neurodegenerative, defined by location, timing, and duration. Inflammation outside of the CNS involves the infiltration of circulating monocytes and other immune cells, whereas inflammation within the CNS is usually (but not always) independent of peripheral inflammatory infiltration and involves resident glia, such as microglia and astrocytes [41]. Neuroinflammation in neurodegenerative disease was always assumed to be merely a response of the system to other pathophysiological events. However, emerging data from preclinical and clinical studies across a range of neurodegenerative diseases including AD, PD and Huntington’s Diseases, ALS, and multiple sclerosis, among others, have established that immune-mediated events can trigger and drive pathogenesis [42–45].

Increasing age is associated with increased low-grade chronic inflammation, or inflamm-aging [46] due to dysregulation of immune [47], glia [48, 49], or metabolic homeostasis [50]. In humans, age leads to elevations in circulating inflammatory markers such as C-reactive protein [51] and inflammatory cytokines [52, 53]. Dysfunctional inflammatory responses that occur with aging alone may act to induce or simply aggravate inflammatory events already underway in neurodegeneration. Such dysfunction in immune surveillance (usually conducted by microglia and astrocytes) that occurs with age may be the instigator in triggering prolonged inflammation. In AD, a hallmark of disease pathology is the presence of neuroinflammation in the brain, which appears to manifest as reactive responses by astrocytes and microglia [54]. Elevation in pro-inflammatory cytokines in the brains of AD patients leads to an accumulation of Aβ and Tau plaques which ultimately result in neuronal loss [55–57]. Neuronal injury due to accumulating Aβ exists in a perpetuating cycle whereby production of inflammatory cytokines causes release of neurotoxic Aβ, which in turn triggers reactive microglia to release more pro-inflammatory cytokines [56, 58]. In AD, microglia are the primary cell type that engulfs and proteolyzes neurotoxic Aβ [3]. Since Aβ plaques are difficult to break down, the efficiency of the microglial clearance dissipates with time leading to increased amyloid and enhanced release of pro-inflammatory cytokines [57]. As such, microglial responses are likely neuroprotective in the early stage but neurotoxic in the late stage of AD [59].

In humans, inheritance of the apolipoprotein E ε4 (*APOE4*) allele strongly increases the chance of developing AD [60]. The reactive response of microglia and astrocytes in the brain is increased in human patients and mouse models expressing the *APOE4* allele. APOE4 alters the baseline pro-inflammatory response even in the absence of disease, suggesting that APOE4 may indeed cause dysfunctional inflammatory responses that trigger neurodegeneration [61–63]. Furthermore, APOE4 is correlated with dysfunctional microglial clearance of Aβ [64]. Although the majority of people carrying the *APOE4* genetic variant have an enhanced predisposition for AD, the effect size is lower or absent in populations of people with African ancestry compared with Europeans or Chinese [65]. For example, some South American non-industrialized populations appear to benefit from APOE4 in order to survive parasitic infection in early childhood, with no apparent adverse AD-associated effects in aged individuals [66].This lack of association of the allele with disease highlights how genetic variation, environmental factors and epigenetics may affect gene-associations of disease.

Similarly, in age-related macular degeneration (AMD) and glaucoma, *APOE4* is protective against the disease [67, 68]. The reason why this inverse relationship is seen in retinal disease and a positive correlation with disease is seen in AD is intriguing. In a mouse model of AMD, mice with the human *APOE4* variant had *less* reactive microglia [69]. Reactive microglia in the retina are already proven to be pathological in glaucoma, so perhaps less-reactive glia in the retina are protective in the case of *APOE4* variants whereas dysfunctional microglia in AD are detrimental. A deeper understanding of evidence across disease pathologies like this that will enhance our understanding of glaucoma as a neurodegenerative disease and will allow us to understand how neuroinflammatory events contribute to disease pathology across the spectrum of human populations.

Not all disease-linked mutations cause direct responses from cells to increase inflammatory mediators. In ALS patients, harboring genetic mutations in the superoxidase dismutase enzyme (SOD1) accounts for about 5% of ALS cases. These mutations do not alter the basal microglial or astrocyte transcriptome, but instead drastically lower the astrocyte threshold to inflammation making them poised to respond faster and more aggressively [70]. Such studies highlight the importance of investigating prodromal and secondary inflammatory responses and functions in cells expressing disease-associated mutations.

In PD, similarly to AD, protein aggregations are a key pathological element; post-mortem examination has identified aggregations of α-synuclein in Lewy bodies of patients with the disease [71]. These protein aggregates that accumulate in the neurons of the substantia nigra are unable to be cleared, triggering neurodegeneration. Since the discovery of high numbers of reactive microglia in postmortem brain tissue of PD patients, it has been suggested that neuroinflammatory events could be the initial instigator of pathogenic mechanisms in PD [72]. Like dysfunctional neuroinflammatory mechanisms in AD, the same “missing-link” question can be posed for PD: are neuroinflammatory events responsible for misfolding of proteins, i.e., triggers of the disease, or are they secondary to protein aggregations? Interestingly, there have been studies correlating the use of non-steroidal anti-inflammatory drugs (NSAIDs) with the prevention or delay of PD [73]. Similarly, the glucagon-like 1 peptide receptor agonist, NYL01, originally developed to combat inflammation in diabetes, has proved beneficial in limiting microglia cytokine release and astrocyte reactivity in mouse models of PD [74], as well as in the bead-occlusion model of glaucoma [75]. These findings highlight neuroinflammation and systemic immune responses as active contributors to progression of disease and the importance of understanding crosstalk between the CNS, PNS and vascular system in disease. Below we discuss the role of additional factors such as mitochondrial pathology, in diseases such as PD.

While these results suggest that NSAID reduce systemic inflammation associated with PD progression, they do not resolve why, in general, anti-inflammatory therapy for neurodegenerative diseases often ends up fruitless. Indeed, anti-inflammatory or antioxidant therapies for neurodegenerative diseases in clinical trials have often been disappointing. An important factor in the role of neuroinflammation in neurodegeneration is timing. It is possible that neuroinflammatory responses have a time and a place for beneficial effects, yet drastic detrimental effects when activated and persisting at the wrong time in disease.

Until recently glaucoma was not considered an inflammatory disease largely due to the supposed immune privilege state of the retina arising from the blood-retinal-barrier (BRB). However, there is accumulating evidence to the contrary in studies from both animal models of the disease and in human patients [76, 77]. Most of our understanding of how the immune system responds in glaucoma has been derived from animal models where onset of elevated IOP leads to early and almost immediate increase in microglial activation and reactivity [78–83]. In post-mortem tissue from human patients, reactive microglia in the ONH are evident [84, 85]. Inflammation in glaucoma appears to be paradoxical; there is a basal level of intrinsic immune surveillance and reactivity that is required to maintain homeostasis, which can even stimulate regeneration (see below) and yet, too much stimulation of inflammatory pathways is associated with degenerative events. In the retina and optic nerve, resident glia (microglia, astrocytes, and Müller glia) act as the immune surveillance and maintain homeostasis by clearing cellular debris, releasing neuroprotective factors, and maintaining homeostasis [86, 87]. A sudden insult, such as an increase in IOP can tip the balance and trigger resident glia to adopt a reactive pro-inflammatory, degenerative state. In addition to resident immune surveillance, there is clinical evidence of transient optic disc microhemorrhages in patients independent of IOP, indicating a clear breach of the blood-retinal-barrier (BRB) and infiltration of circulating immune cells that are associated with disease progression [88–94].

The infiltration of circulating immune cells through BRB rupture may also lend some explanation to an autoimmune component of the disease seen in animal models and human patients [95]. Serum auto-immunoglobulins against heat-shock proteins (HSPs) have been found in the retina of animals and humans with glaucoma, and inoculation of rodents with HSP60 and HSP27 induces optic neuropathy [96, 97]. A link between IOP elevation, intact commensal microflora, and T-cell activation may in part explain HSP-derived autoimmune reactivity. Gut microbiome-sensitized CD4^+^ T-cell infiltration into the retina promotes the progressive degeneration of the retina and optic nerve after microbead-induced IOP elevation [98]. After IOP insult, T cells specifically reactive to HSPs infiltrate the retina; germ-free mice did not show any evidence of neurodegeneration after IOP elevation [98]. These results provide evidence that T cells reactive to host microflora mediate prolonged degeneration of the optic nerve after injury.

How circulating immune cells affect resident glial responses and to what extent factors released by these cells encourage neurodegeneration remain uncertain. It is possible that infiltrating cells could promote regeneration of cell processes lost by acute retinal inflammation. In the PNS, the innate immune response to injury plays an essential role in enabling sensory and motor neurons to regenerate axons back to their peripheral targets [99]. Interestingly, a spike in IOP can also cause an initial influx of macrophages and neutrophils that express molecules (e.g., oncomodulin and SDF1) that can initially stimulate growth of the axon [100–103], leading to the questions of what determines cellular release of pro-regenerative molecules vs. pro-degenerative molecules under stress conditions and whether there are cells that can be coaxed towards pro-regenerative states through release of specific inflammatory factors. In glaucoma, involvement of the inflammatory response in disease progression is indisputable, but more research into the pleiotropic role of immune cells is warranted.

Increasing knowledge of the role of astrocytes and microglia in disease has led to the identification of a pro-reactive sub-state of astrocytes (triggered by reactive microglia) that play a key role in driving retinal degeneration by release of toxic lipids [104, 105]. Astrocytes have been identified as important early responders to unilateral IOP elevation and optic nerve injury by redistributing metabolic resources to the site of injury to promote optic nerve health [106]. Understanding how reactive astrocyte sub-states can drive disease states, or play protective roles, is fundamental to advancing our understanding of inflammation in disease.

#### Metabolic stress

The energy produced by mitochondria (in the form of adenosine triphosphate; ATP) is required for synthesis of neurotransmitters, bidirectional axonal transport, restoration of ion gradients, buffering of calcium and the organization of synaptic vesicles, among other functions [107]. Mitochondria are highly dynamic organelles, and continuously change their size, shape, number, and cellular location to meet metabolic demands of neurons. In addition, mitochondrial fusion, and fission are important for the inheritance of mitochondrial DNA. There are several important processes that mitochondria can undergo to meet metabolic demands; however, they can become dysfunctional in disease [108]. Mitochondrial biogenesis describes the biosynthetic process of increasing mitochondrial number [107], while a delicate balance between fusion and fission allows for the rapid adaptation to meet metabolic demands [107, 109]. Mitophagy, or mitochondrial degradation and clearance is also imperative to maintain cellular homeostasis. Finally, mitochondria are transported along the length of neuronal axons to synaptic terminals and dendrites to provide energy at different focal locations along the neuron 107.

Besides the inheritance of genes that can cause mitochondrial disease, increasing age increases spontaneous mutation of mtDNA [110]. Aging can also cause mitochondria to function less efficiently, which results in elevated production of reactive oxygen species (ROS), that in turn can trigger further mtDNA mutation, pro-inflammatory signaling, and protein dysfunction. ROS production is an unavoidable byproduct of aerobic respiration along the electron transport chain, and complexes I and III account for up to 90% of cellular ROS production [111]. Although ROS are important for cellular signaling, an imbalance leave mitochondria dysfunctional and less efficient at producing ATP. In addition, ROS can cause lipid peroxidation in cell membranes, leading to droplet accumulation in glia a process that is exacerbated in neurodegeneration [112, 113].

Mitochondrial dysfunction has been linked to PD, based on the discovery of the roles of PTEN-induced putative kinase 1 (PINK1) and parkin (PRKN) in mediating mitochondrial mitophagy [114]. Mutations in PINK1 (*PINK1*) and PRKN (*PARK2*) genes were among the first genes to be linked to autosomal recessive PD [115, 116], and there has been increased focus on mitochondrial roles of inherited gene mutations in PD [117]. For example, *LRRK2* mutations lead to α-synuclein aggregates on the mitochondrial outer membrane [118, 119]. It should be noted that PD-associated genes *PINK1* and *LRRK2* are highly enriched in astrocytes over other CNS cells [120, 121] – again implicating non-neuronal cells and inflammation in the pathogenesis of this neurodegenerative disease.

Impaired energy metabolism and defects in expression of genes related to mitochondrial bioenergetics are commonly associated with characteristics of AD pathology [122], including altered mitochondrial biogenesis, mitophagy, fusion/fission and axonal transport of mitochondria [122]. For example, Aβ aggregates cause increased ROS production that can activate downstream proteases that act on mitochondrial fission/fusion GTPases [122]. In the case of mitochondrial transport, Aβ associates with motor machinery including kinesins [123] and dyneins [124]. In glaucoma, evidence of mitochondrial dysfunction is commonly associated with RGC degeneration. Abnormal mitochondrial morphology and distribution has been noted in humans and animal models [125, 126]. In a model of murine glaucoma, mitochondrial transport in RGCs (including number of transported mitochondria, distance transported, and rate of transport) is affected both in the early and late stages of the disease [127]. Furthermore, aged mice exhibit differences in mitochondrial transport and are more susceptible to elevated IOP-driven changes than young mice [127]. Elevated IOP also affects mitochondrial bioenergetics in the visual cortex of the brain in rats; ATP production was reduced, superoxide production was increased and differential mitochondrial complex activity was observed [128].

More generally in neurodegenerative conditions, mitochondrial transport might be hijacked to communicate a stress signal after a local lesion or infarct. Conversely, the movement of mitochondria could be harnessed therapeutically for viral delivery or to promote increased clearance of waste products in disease. When mitochondrial dynamics are altered, either through dysfunction or genetic mutation, the impact for neurons can be catastrophic. The retina is one of the most metabolically active tissues and requires precise regulation of energy supply to meet demands [129]. The unmyelinated portion of the RGC axon in the retina lacks saltatory conduction and therefore is less efficient generating action potentials [107]. Since RGCs rely heavily on mitochondria in the unmyelinated segment, dysfunctional mitochondria lead to optic neuropathies that result in vision loss. Many of these optic neuropathies occur through the inheritance of a specific genetic mutation. For example, mutations in Optineurin (*OPTN*) affect mitophagy and these have been linked to incidence of glaucoma [130]. Mutations in the *OPA1* gene affect mitochondrial fusion and leads to dominant optic neuropathy, the most common inherited optic neuropathy [131]. Mitochondrial DNA (mtDNA) can also harbor mutations that lead to disease, including Leber’s Hereditary Optic Neuropathy (LHON), which can occur due to a mutation in any of several mtDNA genes [132, 133].

#### Neurovascular coupling

The metabolic demands of the CNS necessitate a tightly controlled supply of nutrients and metabolites to maintain cellular homeostasis. Neuronal activity (i.e., metabolic demand) and blood flow (i.e., metabolic supply) are coupled such that an increase in neuronal activity evokes increased blood flow to the area [134]. This neurovascular coupling is mediated by multiple cell types that together comprise the neurovascular unit (NVU) [135], including vascular smooth muscle cells, pericytes and endothelial cells as well as astrocytes, microglia and oligodendrocytes [136–139]. Aside from metabolic support and waste removal, a major role of the NVU is to maintain the integrity of the blood-brain-barrier (BBB), which mediates controlled communication between the CNS and the periphery [140, 141]. The BBB protects the CNS from the systemic circulation and regulates the transport of serum factors and neurotoxins, which could perturb homeostasis [142]. The BBB is not passive; the presence of specialized tight junctions and transporters on luminal and abluminal membranes along with membrane-bound enzymes make it a highly selective and metabolic site of exchange [143]. A specialized CNS glymphatic system involving cerebral spinal fluid, interstitial fluid and lymphatic vessels contributes to the exchange of nutrients and signalling molecules with clearance products such as proteins and solutes in the brain parenchyma [141]. Recently, an ocular glymphatic system was described as an eye-to-cerebrospinal fluid (CSF) pathway that supports clearance of waste products from the retina and the vitreous [144].

The function of the BBB and glymphatic systems of the brain and ocular tissues are fundamental to neuronal health and have implications in the progression of neurodegenerative diseases. Some 30% of dementia patients are specified as suffering from VCID, which represents the second most common cause of dementia after AD [145, 146]. VCID arises from stroke or other vascular injuries that cause significant changes to cognitive functions. VCID shares comorbidity with other common dementias such as AD. Around 60% of AD patients show significant signs of VCID [145], and VCID may involve impaired clearing of Aβ, which is also observed in AD patients [147]. Neurodegeneration also involves a compromise or breakdown of the NVU, which can arise from the disruption of astrocyte connections with blood vessels [145]. Increased reactivity of astrocytes and microglia leads to changes in morphology that can destabilize the NVU and compromise the BBB, which initiates of a pro-inflammatory and pro-degenerative cycle involving peripheral immune system invasion.

A risk factor for AD, APOE may be protective of the peripheral vascular system, along with other molecules such as high-density lipoprotein (HDL). There appears to be a functional interplay between lipoproteins and how they modulate the vascular system, and in turn their indirect effect on neurons in the CNS. APOE peripherally associates with HDL and has been linked to clearance of Aβ in vitro [148]. While HDL and APOE work together to help transport beta-amyloid into vessels, the ApoE2 isoform is more effective than other forms of APOE [148]. Thus, HDL could be neuroprotective target in amyloid-driven disease, as could APOE in the clearance of α-synuclein in PD.

In glaucoma, although a vascular theory of the disease has generated some debate over the decades [149–152], the role of cells in the neurovascular unit in the disease is only recently becoming clear [32, 153]. Glaucoma involves alterations in the vasculature, both morphological (i.e. blood vessel diameter, capillary dropout) and functional (i.e., NVC dysfunction) [32]. Neurovascular coupling in the ONH and retina has been elegantly demonstrated through measurements of hemodynamic responses to flicker-light stimulation [154–157]. In glaucoma patients, flicker-light induced retinal vasodilation is diminished [158, 159]. Interestingly, short-term acute IOP elevations do not alter flicker-light responses, suggesting diminished responses in glaucoma are not due to changes in IOP alone [155]. This evidence hints at underlying dysfunction in the NVU, either due to reduced neuronal activity or altered glial cell function [160, 161].

Recently, an important role for pericytes in coordinating NVU responses in the retina has been highlighted as an integral component of RGC homeostasis and function [153]. Pericytes are highly mobile and interact to finely tune blood flow through capillaries in the retina through inter-pericyte tunnelling nanotubes (IP-TNTs), as visualized though in vivo imaging [153]. Pericyte IP-TNTS are a key component of microcapillary blood flow regulation and are damaged in ocular hypertension [162]. This work highlights not only a potential role for dysfunctional pericyte networks in neurodegeneration, but also the accessibility of the retina as a model for CNS disease. In addition to neurodegeneration of the retina, a pathogenic role for APOE4 in pericytes has also been shown in an in vitro model of cerebral amyloid angiography, reiterating the important role of pericyte function in neurodegenerative disease [163]. Understanding how pericytes react in retinal disease could inform mechanisms of neurodegeneration in AD, PD and traumatic brain injury.

#### Genetic contributors

Characterization of genes responsible for neurodegenerative diseases allows at least partial understanding of risk through inheritance of disease-associated alleles, and thus heritability is often used as a population-based measure of risk for developing a particular disease. Heritability is formally defined as the proportion of phenotypic variance due to genetic factors, although it does not mean that inheritance of a gene will cause disease, and similarly not all individuals with the disease will carry the same risk alleles. Progressing from heritability to disease mechanisms is not a trivial task. One important question to consider is whether the risk allele resides in a gene directly affecting disease, e.g., is it monogenic in nature (a “core gene”), or whether it is a mutation in a “peripheral gene” only indirectly affecting the course of disease through potential regulation of or interaction with core genes [164]. Although genome-wide association studies (GWAS) have identified novel single nucleotide polymorphisms (SNPs), these have generally not been useful for generating disease risk predictive models for use in the clinic [165]. One major reason for this is that many neurodegenerative diseases are polygenic in nature [166]. A better determination of genetic risk of developing disease is through the compilation of a polygenic risk score (PRS). The score considers the small effects of many genetic variations that contribute to disease risk, better capturing the polygenicity of a disease. Indeed, capturing the polygenicity of a disease may lead to the identification of co-morbidities between diseases and common mechanisms to combat more generally a broad range of neurodegenerative diseases.

Genome-wide association studies have been critical for identifying risk factors in AD [164] and studies have highlighted common gene-linked pathways e.g. *APOE4* and the closely associated lipoprotein *CLU* [167]. As noted above, *APOE4* is a shared risk factor for both AD and Parkinson’s disease dementia (PDD), and there is evidence for an APOE-genotype effect on multiple aspects of protein aggregation, inflammation, and neurodegeneration across several distinct diseases including AD and PDD [168–171]. Studies that have combined genetic risk factors across diseases in mice have provided an insight into the mechanisms linking APOE genotype to other neurodegenerative disorders. Transgenic mice that develop alpha-synuclein pathology (Lewy bodies) have been genetically crossed to genetic isoforms of the *APOE* gene [172, 173]. *APOE2* genotype protects against alpha synuclein degeneration compared with other *APOE* genotypes while *APOE4* genetic background had the highest burden of alpha synuclein pathology [172]. These results raise the questions of whether the effects of the protective *APOE* genotype are executed at the gene level or at the level of protein, which has ramifications for leveraging genetics to create neuroprotective gene replacements. Like many genes that putatively harbor disease-associated mutations *APOE* is enriched in astrocytes and microglia.

The effect of sex differences on neurodegeneration is intriguing and highly complex. In the CNS, sex differences are generated by both long- and short-term epigenetic changes caused by gonadal hormones and their interaction with transcriptional gene products found on sex chromosomes [174, 175]. Sex hormones and sex chromosomes therefore each play a part in the response of the CNS to diseases and aging [174]. Aging and disease are both associated with changes in levels of hormones, such as testosterone, estradiol, progesterone, and downstream neuroactive metabolites [176]. Primary examples of changes in levels of hormones are in pregnancy or during menopause with both affecting the process of brain aging in females [177].

Of the studies that have focused on sex differences in neurodegenerative disease, many have highlighted a clear role of differences between male and female biology in disease progression. In these studies *APOE4* increases the risk of AD to a greater degree in women than in men [178], women are less likely to recover from stroke than men [179], estrogen has proven neuroprotective effects in females [180, 181], and sex differences exist in the use of cholinesterase inhibitors for the treatment of AD [182]. Interestingly, sex-driven pathophysiological changes in neurodegenerative disease have also been linked to glial cell populations [174]. Indeed, the sex chromosome complement determines differences in transcriptional responses in glia in response to injury or disease [174]. Furthermore, downstream metabolites of gonadal hormones can interact directly with hormone receptors on many types of glial cells to elicit specific neuroprotective responses [174, 183]. As well as possible direct effects of sex hormones on neuronal health, sex hormones can also affect the vasculature which indirectly affects neuronal survival. The role of sex hormones in maintaining the integrity of the BBB has been recently reviewed [184]. Moreover, the vasculature in the can generate sex hormones locally [185]. Sexual dimorphisms are also abundant in glaucoma; there is increasing evidence that lifetime exposure to estrogen may alter the pathogenesis of glaucoma and that estrogen may have a neuroprotective effect on progression of POAG [186, 187].

Over the last decade, genetic studies including GWAS have identified over 260 risk alleles for glaucoma. Studies of heritability of disease have shown that glaucoma, specifically POAG, is one of the most commonly inherited diseases [188]. Family-based linkage analyses have identified three monogenic risk genes for the disease: *MYOC, TBK1* and *OPTN* [188]. Monogenic risk factors, however, only account for less than 5% of all cases of POAG, suggesting that risk factors for the disease are polygenic in nature; high heritability is due to hundreds or maybe even thousands of gene variants with an additive effect on disease inheritance. Many of the risk factors that have been identified are still related to IOP, thus there is a need for larger patient cohorts to identify additional risk alleles for the disease [189]. The polygenic nature of the disease makes discovery of single-gene loci less impactful. To date, there has not been a monogenic-based gene therapy for glaucoma in clinical trials. Numerous possible applications of gene therapy targets include increased aqueous humor drainage for long-term IOP stabilization, inhibition of fibrosis following filtration surgery, modification of scleral biomechanics for IOP tolerance, RGC neuroprotection and neuro-regeneration, and inhibition of inflammation [190, 191].

In a very recent, high-powered PRS study using glaucoma patient data from United Kingdom, Australia and the United States, the investigators were able to predict glaucoma susceptibility and progression [166, 192]. Using the PRS enabled the detection of patients in the early stage of disease who were particularly high-risk and detection of lower risk patients who could undergo a less-intensive monitoring strategy. The PRS strategy for glaucoma is pioneering in its approach to identify patients who may benefit from potential neuroprotective treatment and represents the first step towards personalized medicine decision-making for glaucoma.

### Detecting and tracking neurodegeneration

Once triggered, symptoms of neurodegeneration may not be apparent until late in progression. Providing patients with a neuroprotective treatment prodromally or early in disease requires definitive early biomarkers, a prospect limited by our understanding of molecular events in disease progression, the pleiotropic nature of most degenerative diseases, and the ability to detect putative biomarkers with sufficient sensitivity. As well, there is a certain level of heterogeneity in clinical presentation from patient to patient. For example, in glaucoma, measurable outcomes such as minor visual field deficits, optic cupping and IOP readings in early disease can be easily missed and are highly variable and perhaps less reliable [193, 194].

Although genetic risk factors can inform clinicians of high-risk patients, carrying a disease allele does not necessarily imply disease. Identifying shared biomarkers early in progression across a range of neurodegenerative diseases will enable very early detection of changes at the molecular level before neurodegeneration occurs, providing a larger window for therapeutic intervention. The term biomarker has been defined by the National Institutes of Health as “a characteristic that is objectively measured and evaluated as an indicator of normal biological processes, pathogenic processes, or pharmacologic responses to a therapeutic intervention” [195]. The objective of identifying new biomarkers for clinical and therapeutic research is to provide a readable output that is robust, reproducible and reliably able to report on clinical outcomes in disease, i.e. is able to provide a reliable prediction of disease onset, progression, prognosis or outcome after therapeutic intervention [195]. The identification of subgroups of patients with specific biomarkers may lead to the identification of the most effective therapies [196]. Furthermore, biomarker-targeted therapies may be more efficacious at different time points in disease. Early biomarkers for disease detection are therefore urgently needed.

#### Biomarkers for disease

**Fig. 2:**
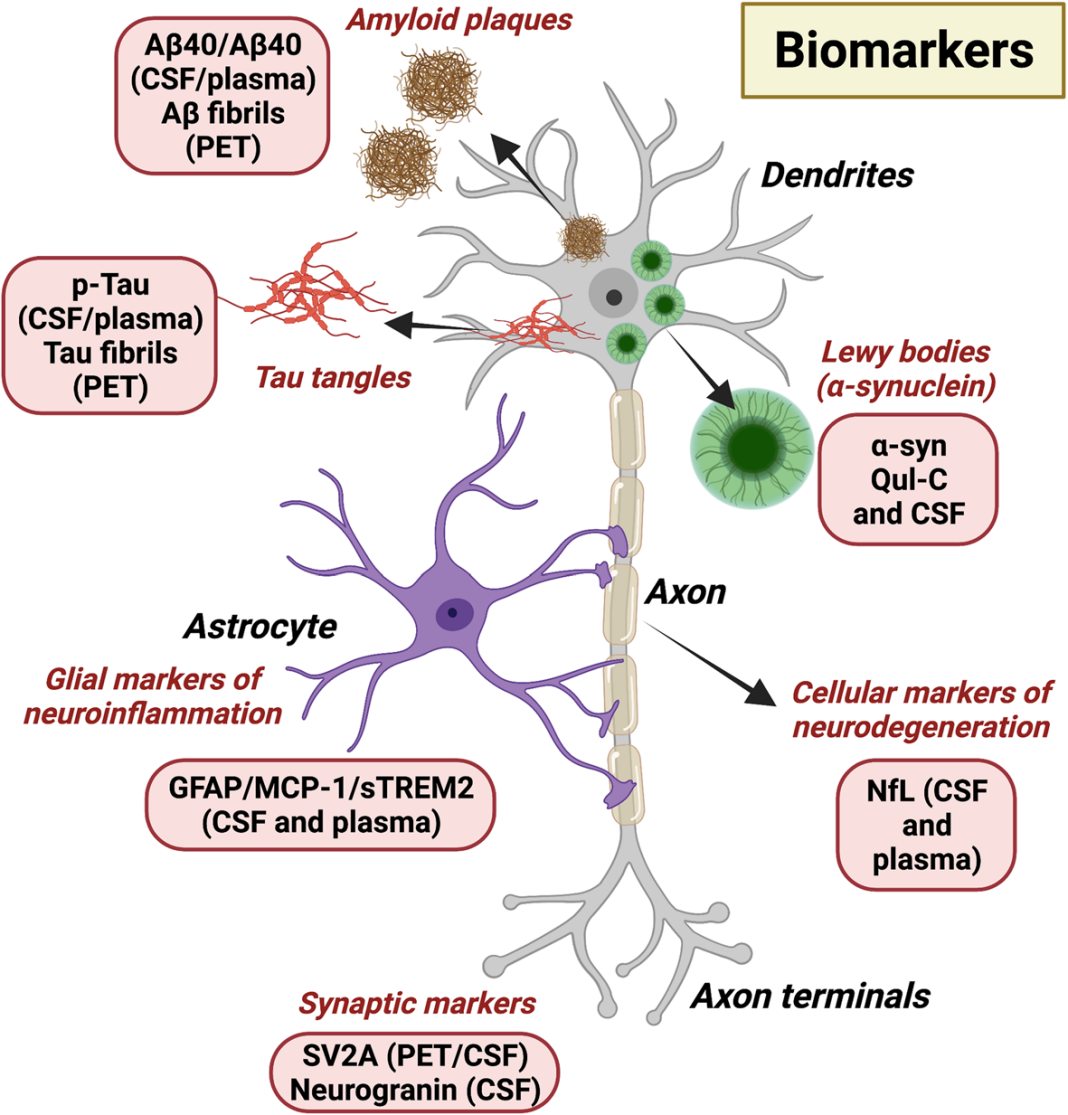
Biomarkers for neurodegenerative diseases. Numerous biomarkers for neurodegeneration are being developed. Amyloid pathology in AD can be readily detected in plasma by measuring the Aβ42/ Aβ40 ratio. Alternatively, larger Aβ plaques and fibrils can be detected visually by Aβ-PET. Similarly, tau pathology can be detected as p-tau in plasma and cerebrospinal fluid (CSF), and tau plaques can be identified as fibrils on PET. Lewy bodies, composed of misfolded α-synuclein (α-syn), can be detected in CSF of PD patients or by using α-syn seeding assays such as α-syn RT-QuIC. Neurofilament light protein (NfL), a marker of degenerating myelinated axons is detectable in CSF and plasma. Several novel emerging biomarkers include neurogranin, a marker of post-synaptic degeneration and synaptic vesicle 2 A (SV2A), a pre-synaptic marker of degeneration. In addition, the presence of reactive gial cell markers (e.g., glial acidic fibrillary protein; GFAP, monocyte chemoattractant protein-1; (MCP-1) and Triggering Receptor Expressed On Myeloid Cells 2; TREM2) in CSF and plasma are being explored as novel biomarkers in neurodegeneration

Progress in the detection of early neurodegenerative disease biomarkers in biological fluids, such as CSF, saliva, and blood has advanced dramatically. These advances have been reviewed in detail recently [196, 197]. Potential fluid biomarkers that fall under the main pathophysiological aspects of neurodegeneration including blood tests, protein aggregates, neuroinflammation markers, and cell death markers have been characterized for many diseases [197]. Primary targets for detection include biomarkers of Aβ pathology, tau pathology, α-synuclein pathology, proteins associated with neurodegeneration, and markers of glial reactivity, for example GFAP [196] (Fig. 2). In addition to fluid biomarkers, high-powered neurological imaging has proved to be a potentially powerful tool for detecting early manifestations of neurodegenerative disease. Imaging modalities currently include magnetic resonance imaging (MRI) and positron emission topography (PET) analysis [198]. Novel PET ligand portfolios for specific neurodegenerative diseases, combined with structural analysis using MRI have enabled the further understanding of temporal changes in neuronal tissue in degenerative neurological disorders. However, there is an absence of cell sub-state specific imaging ligands to offer high fidelity imaging to track disease progression or effectiveness of therapies non-invasively in patients.

In CSF or plasma, the ratio of Aβ42/ Aβ40 reflects Aβ pathology in the brain of AD patients; the levels of CSF Aβ42, but not Aβ40 decrease by up to 50% in AD patients [199]. The detection of changes in CSF fluid Aβ levels are found earlier than PET-detection in the brain but correlate well with PET results [199, 200]. Indeed, a recent publication with head-to-head comparison of eight plasma amyloid-β 42/40 assays in AD showed that the PrecivityAD™ CLIA-approved mass-spectrometry-based blood test performed better when predicting brain pathology [201]. Tau pathology is another protein readily measured in AD patients through PET imaging or fluid analysis. Several PET ligands, specific only for insoluble Tau fibrils in AD brain tissue have been implemented in AD diagnostics [202, 203]. Other markers of neurodegeneration include Tau post-translational modification markers [204], which are suggestive of myelinated axon degeneration. In PD, misfolded α-synuclein is reliably detected in CSF, with levels decreased in PD patients [196]. Other methods for sensitive detection of misfolded prion-like proteins have been implemented including cell-free seeding assays (e.g., α-syn-QuIC) [196]. Seeding assays use CSF samples to detect pathological aggregations of protein and preliminary experiments indicate that the technology could be done using non-invasive skin biopsies [205, 206].

To detect widespread neurodegeneration in the brain, structural magnetic resonance imaging (MRI) is used. MRI allows accurate determination of temporal changes in gray and white matter volumes and such studies have been carried out longitudinally in clinical trials [207]. One drawback to MRI is that it does not allow the detection of specific cell populations that may be particularly vulnerable to neurodegeneration. High-resolution protein-specific methods, such as PET, have correlated changes in specific synaptic proteins, such as synaptic vesicle 2 A (SV2A), with AD and PD onset and progression [208, 209]. Additional fluid detection of markers associated with neurodegeneration include neurofilament light protein (NfL) [210], which shows the presence of brain injury in a number of neurodegenerative diseases, autophagosomal and lysosomal markers as indicators of cell degeneration [211], and neurogranin, a marker of post-synaptic degeneration [196]. Neuroinflammation is a common mechanism across neurodegenerative diseases and there is an increased interest in examining neuroinflammatory markers as indicators of early disease detection and progression. For example, the presence of active glial cell markers such as GFAP, monocyte chemoattractant protein-1 (MCP-1), and sTREM2 in CSF [211, 212]. In multiple sclerosis patients, increased levels of CSF GFAP have been found to correlate with disease severity and progression [213]. In early stages of AD, increased levels of sTREM2 in CSF are detected in patients [212], whereas in late stages of the disease there are increased levels of MCP-1 [212]. Novel markers of cellular degeneration and neuroinflammation may enable clinicians to identify sub-populations of patients at early or late stages of disease for novel therapeutic treatments.

#### Imaging in glaucoma and beyond

The retina, as an extension of the CNS, provides a non-invasive and easily accessible window for high-resolution imaging of CNS tissue. In glaucoma, light-based imaging modalities such as fundoscopy and optical coherence topography (OCT) are more accessible and cost-effective than neural imaging to assess neurodegeneration. RGC degeneration in glaucoma is routinely visualized in the clinic using OCT and presents as thinning of the retinal nerve fiber layer [214]. Retinal vasculature can also be readily visualized using OCT-angiography (OCT-A) and fluorescein angiography and provides the ability to detect microvascular changes early in glaucoma progression. Recent advancements in imaging technology in the eye in conjunction with fluorescent annexin A5 has enabled scientists to detect degenerating RGCs by DARC (Detection of Apoptosing Retinal Cells) in mice and in humans [215]. DARC has moved into clinical trials with patients and is a method well-tolerated, although DARC is currently used as an exploratory endpoint in disease [216]. Although future methods like DARC may aid in detecting populations of patients that have a rapid rate of disease progression, earlier visual biomarkers for glaucoma are critically needed to detect disease before apoptosis of RGCs is triggered.

Imaging of the retina for biomarkers is not exclusive to diseases of the visual system such as glaucoma. In fact, biomarkers for neurodegenerative diseases that primarily affect the brain have also been detected in the neural retina [217]. There are two plausible mechanisms by which neurodegenerative markers may be present in retinal tissue as well as in the brain. The first possibility is that manifestations of neurodegenerative disease in the brain are also concurrently appearing in the retinal tissue. In the aging retina, deposition of aggregated tau, α-synuclein and Aβ are detected [218]. In AD and PD patients the same aggregations are also observed in the retina [219, 220] which suggests that the protein aggregations may mediate neurotoxicity to RGCs in the same manner as neurons elsewhere in the CNS. The second mechanism may occur due to alterations in the brain with neurodegenerative pathology that cause retrograde degeneration of RGCs [217, 221]. In AD, in vivo studies using OCT have found reduced retinal layer thickness [222–225], and reduced microvascular density [226, 227].

Detection of early biomarkers for other neurodegenerative diseases in the eye raises the possibility that the eye could be used as a window to the CNS to monitor biomarkers for neurodegenerative disease in general. There are many benefits to visualization of biomarkers in the eye. Firstly, the process can be minimally invasive and easily accessible. The ability to quantify meaningful molecular biomarkers streamlines patient cohorts for putative clinical trials, reducing noise and enabling smaller, more powered clinical trials [197]. However, systems to visualize pathologies in the eye are limited not only by technological limitations and generating high resolution images, but also by the analysis of such images and lack current understanding about the pathophysiological role of the biomarkers being targeted.

### Model systems for testing therapeutics

**Fig. 3:**
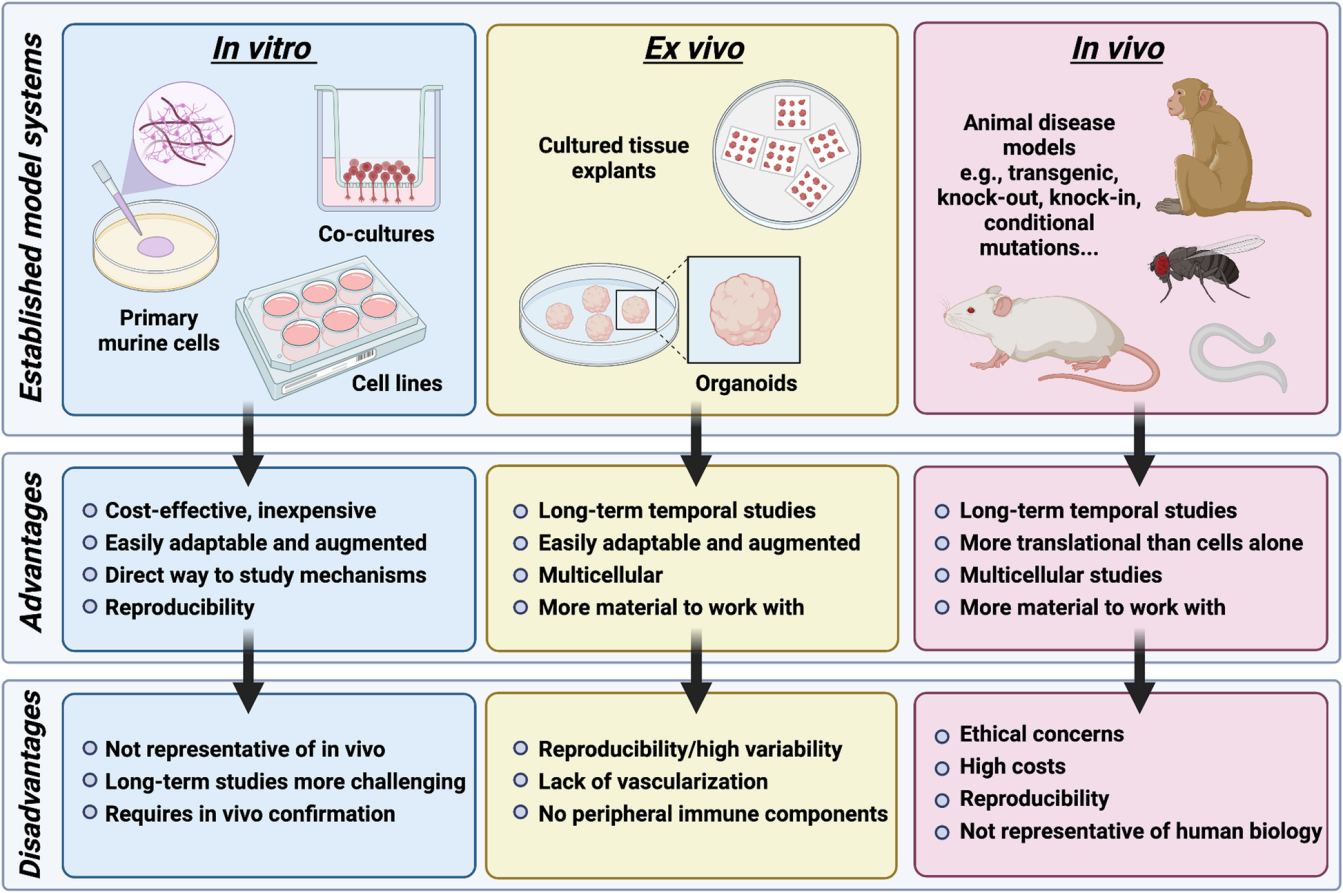
Model systems for studying neurodegeneration. Established experimental models of disease can be categorized into three main areas: in vitro, ex vivo and in vivo. Each type of model system has advantages that can be leveraged to explore disease mechanisms; however, disadvantages exist for each avenue. In vitro models such as cell lines and purified primary cells are a rapid and inexpensive way to explore disease mechanisms, however, extrapolation of results to biological systems is difficult. Ex vivo models, such as the growth of organoids in culture or explanted tissue cultures are multicellular, allow more complex mechanistic questions to be explored. However, they are not ideal representations of in vivo situations due to lack of vascular or peripheral immune components. In vivo models include animals such as non-human primates, mice and rats, Drosophila and Caenorhabditis elegans, and others. Although these models allow for in vivo studies of disease, the cost is high, and the results may not always translate well to human biology.

The ability to accurately and robustly mimic human disease in the laboratory is key to the success of developing therapies that will translate well into the clinic. However, numerous recent failures in the translation of pre-clinical therapeutics from the bench to beside in clinical trials have raised doubts about the relevance of current animal models for human diseases Current in vitro, ex vivo and in vivo model systems are illustrated in Fig. 3. The etiology of neurodegeneration in human diseases is highly complex, involving multiple cell types, cellular signaling pathways, genetic loci, and environmental cues. Attempts to encapsulate all aspects of a human disease with a single model have not been fruitful. As insights into human diseases grow, translatability of experimental models is an important consideration for the design of novel therapies. For example, mouse models of AD which have been broadly based on human genetic studies, accumulate Aβ but do not develop other common pathologies such as neurofibrillary tangles [228]. Aβ therapy, primarily designed to inhibit Aβ production, aggregation or enhance Aβ clearance, was largely successful in mouse models of AD but did not translate in human clinical trials [229, 230].

Failure in translation of mouse models to humans drives home the point that mouse models may not be ideal for the development and design of human therapeutics. In many cases, the focus of therapeutic intervention is on neuronal populations, while other cell types are not always considered. In AD, the role of vascular dysfunction and immune reactivity are widely accepted as reflecting the importance of cell types besides neurons [231]. One of the possible reasons that genetic models of AD do not translate to humans is that, while leading to the degeneration of neurons, they lack the robust glial and inflammatory responses seen in patients [231]. An obvious bridge between rodent models and humans are non-human primates, although, the use of non-human primates comes with additional ethical issues and extremely high costs. Since costs to house and maintain non-human primates are so high, the number of animals used in pre-clinical studies is often low, and perhaps some would argue under-powered.

In glaucoma, inducible models are sometimes used to the extreme [232, 233]. Some models in rodents reach IOP elevations that are not physiologically relevant to the human disease, with acute IOP levels increasing by up to 200–400% [234]. In fact, many patients with glaucoma never present with elevated IOP, and it is clear that other pathological mechanisms are at play. The optic nerve crush model in rodents has developed into a useful tool to study regeneration of RGC axons after injury, enabling a greater understanding of the cellular and molecular mechanisms that drive axon regeneration and RGC survival [235]. Such animal models of optic nerve injury have determined that both cell-intrinsic and extrinsic (i.e., environmental) factors have distinct roles in the potential for RGCs to regenerate. Optic nerve crush studies are also integral to identifying factors that may not be regenerative in nature, but rather are pro-survival in nature. Pro-survival factors may also be key to enabling degenerating RGCs to remain viable long enough to move to a pro-regenerative state.

#### In vitro systems of neurodegeneration

In vitro model systems offer a less expensive, highly adaptable, and augmentable system for the high-throughput investigation of novel mechanisms in disease and the design of therapeutic interventions (Fig. 3). In vitro model systems have grown exponentially in their complexity in recent years. Initially, the use of primary cell cultures and organotypic cultures provided researchers with a means to explore disease mechanisms [228]. An attractive ex vivo model is the use of organotypic cultures of brain slices, whole neural retina or retinal slices. Explanted tissue can be prepared from multiple animals and in some cases human donor tissue and can faithfully represent tissue architecture and cellular structure. Even so, the ability to maintain viability in culture remains notoriously challenging [236]. In the context of glaucoma, with what we now know about pathophysiology at the optic nerve head and the need to produce axons that span the length of the optic nerve, organotypic cultures of whole optic nerve and whole retina would be most relevant but are extremely difficult to isolate and maintain. In addition, organotypic cultures do not provide long enough timeframes for the investigation of disease processes that may occur more slowly, such as chronic inflammation and neovascularization. An ongoing problem in many primary cell culture experiments is the inclusion of serum in media. Serum is largely excluded from the CNS by the BBB, and inclusion in culture experiments irreversibly alters the gene expression profiles and functions of many glia and immune responsive cells like astrocytes [237] and microglia [238, 239]. Similar artifacts can also be induced by the use of enzymes in the digestion of CNS tissue when isolating microglia [240, 241]. Updated methods exist to grow these cells in serum-free defined media [237–239, 242], but they have not been widely applied for unknown reasons. The use of mixed-species multicellular co-cultures has also helped to remove many of the artifacts of serum culture [243].

Human-derived induced-pluripotent stem cells (hiPSCs) from human donors have been useful for generating multiple cell types harboring the same genetic background [228]. This has been particularly important for the study of patient genetics in disease; neurons and other cell types carrying disease-specific genetic mutations can be assessed longitudinally in culture. In early experiments, 2D cell cultures failed to recapitulate cell-cell interactions, and the introduction of scaffolding materials such as agarose and hydrogels has promoted 3D tissue-like structure that better models disease [228]. These innovative reconstructions of CNS tissues may be advantageous when it comes to understanding disease progression.

#### Organoid cultures

Growth and differentiation of hiPSCs in culture has led to cerebral organoid structures that can exist for several months and exhibit similar manifestations of neurodegenerative disease as the human donors from which they were obtained [244, 245] (Fig. 3). Such studies provide the ability to assess the impact of disease genes on physiological processes over time, highlighting key windows of opportunity in the disease progression [246]. In addition to brain, human retinal organoids have been developed with mature photoreceptors that have the ability to respond to light, bringing retinal organoids one step closer to being successfully used for disease modelling, and perhaps even for the regeneration of the retina in patients that have lost vision [247].

A potential problem in the generation of organoid structures, however, is the variability of cell types within cell populations produced when culturing hiPSCs. To better interpret results from organoid cultures, improvement in single-cell characterization is needed. Novel quantitative platforms have recently been developed that may help overcome this issue. These systems have the capacity to analyze human organoids at a single cell level on a large scale to improve quality and reproducibility of organoid structures [248].

One drawback to organoids is the lack of vascular elements, and thus efforts to develop in vitro neuronal-vascular systems are becoming increasingly important as organoid cultures become more complex. Combining in vitro vascular models with multifaceted cellular neuronal circuitry will be pivotal. Also, the BBB and BRB are fundamental to the maintenance of neuronal health and homeostasis in the CNS and are also implicated in neurodegenerative pathology. However, incorporation of vascular elements into in vitro model systems is not simple. In fact, regenerating the multicellular organization of the neurovascular unit is itself a challenge. A very recent study has made a huge step forwards in modelling the neurovascular unit in conjunction with neurons in vitro [249]. The model system utilizes a scaffold-directed approach and multiple cell types, including induced pluripotent stem cell-derived neurons, endothelial cells, astrocytes and smooth muscle cells to generate an in vitro model of an arterial neurovascular unit [249]. Development of this system will increase our understanding of the vasculature in physiological and pathophysiological conditions and may also provide a useful tool in the assessment of novel drug therapies and drug delivery across the BBB to promote neuronal survival. Another drawback to the implementation of organoids in evaluating mechanistic and therapeutic strategies for neurodegeneration is their lack of interaction with the PNS. Infiltration of circulating PNS immune cells is often associated with neurodegenerative disease progression, and organoid cultures do not yet address this potential confound. Alternative strategies such as the implantation of human iPSC-derived organoids into the rat brain to enable vascularization have proven a novel way to potentially overcome this problem [250].

#### A look ahead: new model systems

The advantages of in vitro model systems are twofold for designing new treatments. Patient-derived hiPSC human organoids can be cultivated and analyzed over time so that the disease phenotype of the tissue can potentially be fully characterized over time. This opens the possibility of being able to visualize key changes at various time points in disease progression, and windows of opportunity for novel therapies. Also, in vitro organoids provide a biological system to test promising therapeutic treatments in a potentially more relevant model of disease than mice or cell cultures alone. In view of the flaws in using mouse models mentioned above, and with increased funding for in vitro models, and ethical considerations explored and defined, in vitro model systems could reduce the time and money wasted in the failure of clinical trials by providing a more translatable pre-clinical model.

Human-derived in vitro models are advancing in their complexity and so to overcome the limitations of mice as model systems, mice with human neural transplants can be generated. The brains of these mice are a combination of in vitro hiPSC-derived neural cells engrafted into mouse models, opening up a possible alternative approach to studying the role of specific cell types in disease [231]. Such mice balance the advantages of having a living organism and the translatability of hiPSC-neural cells in one model but raise the issue of ethics of cross-species models. The current consensus is that these models are unlikely to have complex human characteristics, but still raise issues regarding animal welfare that need to be addressed [251].

Although technical challenges are evident in the generation of novel model systems, it is also important to consider the ethical limitations, safety, and interpretation of these exciting new avenues of research. The successful generation of hiPSC-derived organoids raises an exhaustive list of ethical concerns, including informed consent and privacy of cell donors, the potential for organoids to develop human characteristics or qualities, the use of transplantation or even gene editing [252]. The importance of this topic is paramount in the future use of organoids, neural transplants or chimeric model systems for neurodegenerative research [251, 252].

The evolution of human-like organoids and 3D cell culture systems could revolutionize the approach to drug discovery and development, saving money and time and enhancing translatability to human clinical trials. At present, procuring funding for the development of in vitro systems is arguably more challenging than most other model systems, such as mice. One issue is that in vitro model systems are not widely used or easily validated. With increasing studies and improved technologies and ethical considerations, in vitro systems such as hiPSC organoids to model healthy and diseased conditions will facilitate a new era of personalized and precision medical treatment.

### Opportunities for new therapeutics

Across all studies in neurodegenerative disease, a fundamental theme in designing therapies and, ultimately, a cure, is finding the right intervention at the right time. Neurodegeneration is progressive and enhancing our understanding of the temporal aspects of neurodegeneration will inform check points for neuroprotection and regeneration. Neuroprotection relies on the understanding of key molecular changes in tissue as it moves from homeostatic (i.e., healthy) to diseased. The goal for neuroprotective treatment is to provide tissue with the necessary factors to support healthy neurons and to prevent neurodegenerative changes at the molecular level from occurring. A major benefit to providing patients with neuroprotective intervention is that it has the potential to stop the degeneration of otherwise healthy neurons, without the trauma of developing symptoms associated with neuronal death such as cognitive decline in the brain and loss of vision or the challenge of replacing lost cells in the retina.

Patients with increasing cognitive decline due to AD or other neurodegenerations of the brain are patiently awaiting disease-modifying therapies, or therapies that could restore the loss of functional neurons. In addition to people living with end-stage AD-related dementia, a subset of individuals exhibiting pre-symptomatic pathology may benefit from interventional neuroprotective treatment [253]. In line with other areas of the adult mammalian central nervous system, the optic nerve does not have the ability to repair itself after injury. For patients who have lost their vision, restoration may involve complete replacement of lost cells and regeneration of optic nerve axons, or axon-regeneration and rejuvenation of surviving but compromised RGCs [254]. Here we outline the key areas that are providing researchers with the hope to restore cognitive function in patients with neurodegenerative brain diseases or restore vision in glaucoma patients.

#### Leveraging genetics for neuroprotection

With increased understanding of the genetics of neurodegenerative disease comes the opportunity to leverage genetics to inform new treatments. There are two main ways that genetics can be leveraged: (1) through therapy targeted to a causative allele, and (2) by countering the downstream effect of a disease gene pharmacologically. One common disease-associated gene in AD is the microglial gene *TREM2;* gene variants in *TREM2* increase the probability of developing AD by around 2-3-fold [255–257]. Microglia are central to multiple disease pathologies as discussed (i.e., BBB integrity, clearage of waste and Aβ plaques, altering synaptic relationships, reactivity of other glia), and hyper-reactivity of microglia is linked to pathogenesis in neurodegenerative disease. However, human genetics tells the opposite story – all mutations in *TREM*2 lead to decreased functionality [258]. By application of genetics, there is the potential to shift cells, such as microglia, to a pre-degenerative state, potentially rendering them neuroprotective.

Many traits in neurodegenerative disease are genetically correlated in a phenomenon known as pleiotropy, whereby a genetic locus affects multiple characteristics [259]. Identifying potential genetic crosstalk between genes in neurodegenerative pathology has the potential to serve as a therapeutic target for treatments that simultaneously prevent or treat multiple diseases. Identifying high-risk genetic alleles makes possible the road to gene therapy by silencing or replacing disease-causing genes with non-mutant forms. Gene-based diagnostics and screening also enables the identification of individuals at risk for a particular disease before irreversible damage occurs. Identifying patients based on genetic screening can also refine patient cohorts for clinical trials, for example using genetics to define inclusion conditions for a novel drug therapy.

Genetic analysis of neurodegenerative disease can also give rise to potential downstream drug therapeutics. There is an association of more than a hundred loss-of-function mutations in Progranulin (*PGRN*) that cause early-onset dementia [260]. Progranulin is an immune regulatory protein with neurotrophic properties but decreasing the level of PGRN leads to hyperactive microglia and over-secretion of inflammatory mediators, which leads to neurodegeneration. A potential therapy may therefore involve preventing the breakdown of PGRN with drugs targeted specifically to the protein. In this way, genetics has informed us of alternative down-stream pathways that can be targeted in the disease.

The multifactorial nature of AD has been recently highlighted by the development of a multiplex model [261]. AD encompasses genetic mutations in genes across many functionally-distinct molecular pathways; over 50 genetic loci have currently been identified in the development and progression of the disease [261]. This has spurred on the generation of several AD-related mouse models and cell lines, although many of these models focus on single gene effects [261]. Genetic studies have changed our understanding of AD and other related dementias and exploring neuroprotective therapies in the future will rely on assessing multiple gene outcomes in disease models. The challenge of modeling polygenic diseases in animal or cell models is a hurdle that urgently needs to be addressed to create a better understanding of disease mechanisms and to provide treatments that translate well in the clinic.

Since multiple disease pathologies are commonly associated with neurodegeneration, multifactorial disease therapies may prove more effective than monotherapies targeting one aspect of the disease [262]. Combination therapies have been successfully implemented in the treatment of previously life-threatening diseases such as cancer, tuberculosis and HIV/AIDs. Since AD exhibits multiple co-occurring pathologies such as vascular brain injury, Lewy body pathology and TDP-43 inclusions [262], treatment to tackle these pathologies together may show more promise than previous failed attempts, such as clinical trials using anti-Aβ as a monotherapy. An example of such therapy might combine anti-Aβ to promote immune-clearance of Aβ aggregates, with an inhibitor of β-secretase, the enzyme responsible for the production of toxic Aβ [262]. Similarly, glaucoma shares co-morbidity with systemic vascular diseases such as hypertension, and BRB breakdown has recently been highlighted as an important, yet overlooked disease mechanism [32]. Future neuroprotective treatments that combine current IOP-lowering therapies with therapeutics to target novel aspects of pathology such as vascular-targeted drugs or immune-suppressing therapies may be more efficacious than monotherapies in the clinic.

Before effective combined therapies can be offered, however, we need to fully understand the interplay of genetics and progression vs. initiation of disease. Understanding the genetic influence on disease risk will require much larger patient cohorts with combined analyses that includes GWAS, PRS and pathway analysis to better inform studies that aim to identify common genetic risk factors for neurodegenerative disease and leverage them for treatment. In addition, identifying where temporally in disease progression a particular gene exerts its effects is lacking in most studies.

#### The promise of regeneration

With no current cure or effective treatment for neurodegenerative disease and patients progressing to cognitive decline, blindness or even death, neuro-regeneration is the only option to restore otherwise degenerated neurons. For neurodegenerative diseases that primarily impact the brain, such as AD and PD, neuronal loss within cortical and subcortical regions of the brain can be problematic to regenerate due to the potential invasiveness of the procedure required [263]. An ongoing question in neurodegenerative disease is how the peripheral nervous system can regenerate after injury whereas the central nervous system has a very limited capacity for self-renewal and repair. The unique ability of the peripheral nervous system to regenerate after injury has been in part credited to resident Schwann cells [263]. Schwann cells are exclusively found in the peripheral nervous system and have the capacity to drive neuronal repair and axon regeneration after injury through de-differentiation and reprogramming. Re-programmed Schwann cells promote demyelination and secrete neurotrophic factors, growth factors, and other neuroprotective factors to support axon regeneration [263].

In the brain, utilizing elements of the peripheral nervous system, either through grafting or with purified Schwann cells, has shown extraordinary potential in a small number of non-human primate studies, and in human trials in patients with PD, HD and in mice and rats with spinal cord injury [263]. In trials to demonstrate safety of these procedures, patients underwent autologous grafts of peripheral nerves into regions of the brain, or transplants of purified Schwann cells without reports of serious complications and mild improvements in cognitive function [263]. The studies were largely underpowered but do provide some insight into the cells and environment needed to encourage axonal regrowth in the central nervous system. In the central nervous system and by extension the visual system, Schwann cells are absent, but oligodendrocytes fill the role of supporting neurons and myelination of axons. Although some remyelination may occur spontaneously after injury [264], oligodendrocytes generally lack the capacity for regeneration. Oligodendrocyte precursor cells (OPCs) present in the optic nerve, can undergo a transient period of proliferation after injury, however, the response is not sustained, and the cells fail to differentiate into myelination-competent oligodendrocytes [265]. Interestingly, the augmentation of intrinsic OPC signaling through GPR17 coupled with microglial depletion promotes differentiation and the remyelination of regenerated axons, offering a potential *de novo* strategy for remyelination after CNS injury. With advances in stem cell-derived cell types, it may prove feasible that stem cell-derived oligodendrocytes could promote repair and regeneration of the myelinated segments of the optic nerve after injury.

By characterizing the injury response of RGCs after optic nerve crush, several intrinsic RGC-specific factors have emerged with regenerative potential, including deletion of PTEN and SOCS3 or manipulating a variety of transcription factors. In addition, extrinsic factors such as the mTOR-activating proteins such as Osteopontin and several others growth factors have been [254, 266]. By generating a triple deletion murine mutant (*PTEN*^−/−^*/SOCS3*^−/−^*/CMYC*^−/−^) combined with CNTF treatment, lengthy optic nerve axon regeneration after injury was achieved; similar effects have been obtained by combining intraocular inflammation (to elevate Oncomodulin and SDF1) with cAMP elevation and PTEN deletion [266] or by manipulating the mTOR pathway while providing physiological stimulation [267]. Understanding the intrinsic and extrinsic factors that promote outgrowth and survival of RGCs may place us in a better position to coax a regenerative state.

The role of cell extrinsic factors, such as inflammation are also important in regeneration. As in the peripheral nervous system, triggering of an inflammatory response and release of pro-inflammatory mediators can stimulate regeneration of axons. In the eye, lens injury alone is sufficient to stimulate axon growth after crush [268, 269], as are several other pro-inflammatory stimuli [100, 270]. Indeed, in ophthalmic surgery for glaucoma patients, where laser stimulation in some treatments stimulates repair, is it possible then that generating a small amount of local inflammation could encourage reparative growth in the optic nerve? Identifying factors crucial to regeneration of RGC axons is fundamental in generating axons, however, the regenerated axons need to function optimally as mature developed, healthy RGC axons. An important consideration moving forwards in regenerative research is understanding how promoting axon regrowth affects RGC axon function; do factors that promote regeneration also support RGC axon function?

#### Glia-specific therapies for neurodegenerative disease

Multiple and parallel immune cell-astrocyte-neuron signaling axes active during health and disease could provide an exciting possibility for novel drug targeting. What is quite exciting is the commonality of some of these heterogeneous populations across diseases [104], which may provide therapeutic avenues that need not be disease specific. Preliminary investigation into therapeutic targeting of reactive astrocyte sub-states has been leveraged in mouse models of PD where abatement of immune cell dysfunction, and mitigation of astrocyte-induced neuron cell death appears possible using glucagon-like 1 peptide receptor agonists. Such drugs target microglia to minimize astrocyte-reactivity inducing cytokines [74]. This treatment is also reported to produce beneficial outcomes in the bead occlusion mouse model of glaucoma [75]. Other possible therapeutic angles include targeting astrocytes to enhance glutamate re-uptake to minimize glutamate excitotoxicity that is reported in ALS, HD, AD, and other diseases [45, 271, 272]. Other approaches would include global inflammation dampening, or block of specific detrimental reactive astrocyte functions (e.g., production of toxic lipids); or enhancement of other supportive functions like trophic support, synapse formation, or other important developmental functions of astrocytes. For microglia, effective targets would limit pro-inflammatory cytokine release [74] or block toxic metabolite release. This approach is particularly important in patients with mutations that drive additional neuron susceptibility, like the recently reported *Grn*^−/−^ susceptibility in mouse models of fronto-temporal dementia [273]. The same effect could be achieved by enhancing phagocytosis to aid removal of toxic pathogenic proteins – like recent efforts to target TREM2. More holistically, interventions with dietary changes could prove very effective. The recent discovery of peripheral immune cell reprogramming and bacterial load in the gut that in turn cause reactivity changes in microglia and astrocytes, effectively gives an accessible peripheral target for a known astrocyte-mediated neuron cell death pathway in the CNS [274]. Future effective therapies may need to target individual sub-states of reactive microglia or astrocytes to stop the initiation of disease, slow progression of degeneration, or reverse the effects of chronic diseases.

#### Cell replacement strategies to restore vision

Aside from encouraging axon regrowth, there have been some promising studies attempting to integrate retinal cells into animal models of retinal degeneration. Most studies have involved transplantation of either purified photoreceptor cells, retinal pigmented epithelial cells or stem cell-derived photoreceptors into sub-retinal spaces, close in proximity to where the cells are needed to infiltrate [275, 276]. Incorporation of RGCs into the retina is somewhat more challenging, in part due to the likely need for intravitreal delivery and penetrance through the inner limiting membrane [277]. To date, efforts in animal models have been hindered by either lack of integration of replacement cells, or by the capacity of new cells to regenerate axons capable of traversing the distance between the retina and appropriate target cells in the brain.

To improve cell titers and increased likelihood of cell integration into the retina, retinal organoid grafts grown in culture have been implemented in animal models. It was hoped that retinal grafts may increase cell density at the site of integration in the retina leading to greater cell incorporation, yet RGC axons struggled to cross the inner limiting membrane, suggesting that additional factors and/or inner limiting membrane disruption may be necessary to promote cell integration [278]. To improve RGC cell replacement strategies, a large effort to study the development of human-derived retinal organoids in vitro is underway. One challenge to this approach is that human stem cell-derived retinal organoids contain only a small percentage of RGCs and they do not survive long in culture. Learning about the molecules that control the steps through organoid development and manipulating these pathways to generate more RGCs that can survive long-term may prove useful in improving cell incorporation in vivo. Studies on the co-culture of stem cell-derived ganglion cells has shown that co-culture with Müller glia or conditioned media improves survival and axonal growth in culture, suggesting that addition of these factors may help to encourage the transplantation of RGCs [279].

Replacement of RGCs in the retina is not trivial and emphasis on RGC cell type is important when we consider the replacement of functional RGCs in the retina. Recent genetic profiling of RGCs in mice revealed 46 molecularly distinct cell types, which subserve different functions in the visual pathway [280]. Identifying how different RGC subtypes respond to injury at the molecular level may hold the key to harnessing pro-survival factors. In a study of murine models of optic nerve crush injury [280, 281] and ocular hypertension, alpha-RGCs appeared particularly resilient following injury compared with other subtypes [282]. Genetic profiling of RGCs may highlight specific genes that are correlated with resilience and regeneration. Recently published atlases of retinal ganglion cell types in humans provide a starting point for such analyses [283]. Equally important is to understand how distinct RGC types are generated during development, and single-cell transcriptomic analysis of retinal development have begun to provide insight on this subject [284, 285]. Understanding which genes in development promote RGC differentiation might allow us to harness similar pathways for disease.

#### Advances for alzheimer’s disease

Past treatments approved by the US Food and Drug Administration (FDA) have focused on targeting the symptoms of AD, improving cognitive or behavioral functions but not necessarily affecting underlying progression of the disease [286, 287]. This year the FDA carried out an accelerated approval process for the first disease-modifying treatment from Biogen, Aduhelm (aducanumab), an anti-Aβ therapy for the removal of Aβ plaques [288, 289]. The decision by the FDA has been met with scientific controversary [290]. Prior to the FDA’s decision for accelerated approval, clinical trials were halted due to claims of futility, and the data did not meet the rigorous criteria for FDA approval. In the clinical trials that did proceed, over 50% of patients presented with localized brain swelling or microhemorrhages [291, 292]. Despite scientific dispute regarding the efficacy of Aduhelm, production and marketing of the drug will continue in conjunction with a 9-year prospective study requested by the FDA to confirm clinical benefit.

Recently, the concept of resilience to AD pathology or downstream neurodegeneration following pathology have opened up a new avenue of research that may highlight novel targets for disease intervention [293–295]. Resilience to AD has been defined as individuals who exhibit the hallmark neuropathology but no clinical signs of cognitive imparment [296]. Whereas protection from disease is defined in GWAS studies (comparing AD with control subjects) as genetic variants who have a decreased risk of inheriting the disease, a delay in disease onset, or exhibit less pathology than expected [293]. Potential protective targets include variants in *APP* that lead to a reduction in pathologic Aβ [297, 298], *APOE* gene variants including *APOE2 *[251], *APOE3-Christchurch* [299], and *APOE3-Jacksonville* [300] with lower risk of developing AD [301], and variants in cholesterol efflux pathways such as *ABCA1 *[302], amongst others [293]. Identifying potentially protective genetic targets in human populations may bring to the fore core molecular mechanisms that can be harnessed for neuroprotective treatment.

Another novel concept in AD pathology is the possibility that a synergistic pathological interaction exists between Aβ and tau which manifests throughout the course of disease and may drive progression [303]. To date, Aβ and tau proteins have been studied as singular entities in AD pathophysiology. The study of potential synergistic relationships between disease-causing elements requires improved animal or cellular models, integrated with systems approaches such as machine learning to understand such interactions and their spatiotemporal evolution in disease progression.

Therapeutic opportunities that aid in slowing progression or preventing cognitive decline in AD rely on early detection of biomarkers associated with early (or prodromal) neurodegenerative events. Although some advances with blood levels of Aβ and tau have been made recently [253], robust early markers remain elusive in AD. There remains great potential in harnessing the eye for early biomarker detection. The combination of non-invasive imaging using OCT/OCT-A to detect AD-specific alterations in retinal architecture and morphology with the detection of Aβ, tau and neurofilament light chain in the lens, vitreous and retina [304] provides compelling evidence that the eye manifests early AD-related changes that may be non-invasively detected and monitored in patients.

### Opportunities for new models and imaging systems

In drawing upon the common mechanisms of neurodegenerative diseases, we believe that preventing vision loss or preventing neurodegeneration of the brain becomes increasingly achievable. However, identifying and understanding shared molecular mechanisms is only the first step in designing powerful neuroprotective or neuro-replacement strategies. The next steps rely heavily on the validation and rigorous testing of potential neuroprotective or neurorestorative agents, which both involve robust monitoring of RGCs and neurons in the brain. With these goals in mind, several immediate challenges come to the fore.

For major advances in the design and implementation of neuroprotective therapies, the development and characterization of translatable model systems is critical. In human patients, clinical trials of neuroprotective agents are not a viable option; such studies would prove high-risk, expensive and involve extensive, perhaps even decades-long trials without easily measurable outcomes. Without a model system that encapsulates the multifaceted nature of neurodegenerative disease (e.g., including multiple cell types in addition to mature neurons), the development of therapeutic strategies will repeatedly stall.

A new challenge comes with the recognition that neurodegenerative events, as well as tissue homeostasis, are not neuron-centric – they are multicellular in nature and dissecting the roles of multiple cell types is difficult. It is increasingly evident that glia are important for RGC maturation, development, and survival [45, 305]. Understanding how glia affect RGCs and other CNS neurons during development or after injury will be important in designing neuroprotective drugs, but also in promoting integration of replacement cells. Harnessing the properties of other cells may promote RGC/neuron survival or enhance grafting of replacement cells into host tissue. Likewise, glia, in particular microglia, are central to controlling the development of AD pathology and modulating neuronal activity [59]. Understanding how specific microglial responses are protective or detrimental can guide us how to target these cells at different stages of neurodegenerative diseases.

There is also an urgent need for improved imaging systems for use in the clinic and in research as we push forward with neuro-replacement and neuroprotective strategies. Resolution of the retina at the cellular level will be fundamental in the assessment of the efficacy of neuroprotective treatments. One example is the detection of immune cells and assessment of the neuroinflammatory state of the tissue through high resolution imaging. As discussed, neuroinflammation is an over-arching theme in neurodegeneration. Determining immune cell infiltration into the retina, or state of glial cell responses and reactivity would represent a major stride forward for clinicians and researchers alike when trying to tackle neuroinflammation in neurodegenerative disease. Similarly, although a distant milestone at present, monitoring the engraftment of new cells to restore vision or cognitive function will also rely on advanced imaging systems not yet available. Novel high-resolution in vivo imaging modalities will be necessary to achieve these goals. One challenge this presents from a research perspective is the multidisciplinary nature of the expertise required to build high-resolution imaging systems, and as such, a focus in the future on multidisciplinary collaborations across medical and bioengineering fields will be necessary.

## Conclusions

### The challenge ahead

Providing patients with effective strategies to treat or prevent neurodegenerative disease is a monumental challenge that scientists and clinicians alike will increasingly face as the population ages and incidence of disease increases. Reaching these goals will rely on a greater understanding of the common pathological mechanisms across the entire spectrum of neurodegenerative diseases, which include diseases of the brain and by extension, the visual system. Focusing solely on linking molecular mechanisms to a single disease can lead to siloed thinking, inability or unwillingness to make major leaps forward in the development of advanced treatments and cures applicable to the broader scope of diseases.

In this “think tank” style meeting, with multidisciplinary experts from all aspects of human CNS neurodegeneration, we have identified several common molecular mechanisms of disease that highlight the most promising avenues for fruitful collaboration in Table 1. In the diseases touched on in this review, shared mechanisms are manifold, spanning protein aggregation to mitochondrial dysfunction and altered metabolism, to breakdown of neuronal-vascular signaling, just as examples. Work to advance patient treatment and care for neurodegeneration will need not only to address our understanding of the core molecular events that occur but also *when* they occur. We believe that the commonalities among diseases provide new and exciting collaborative research opportunities that we can harness to discover new therapeutics and clinical strategies.

**Table 1:** Key areas of research opportunity in neurodegenerative disease. There are four key areas of opportunity in neurodegenerative research, these include animal and in vitro disease models, neurovascular breakdown, in vivo imaging tools, and biomarker development. The current limitations are outlined and describe the aspects of each research opportunity that need to be overcome to make progress in the design of novel therapeutics. Finally, we highlight the key areas of research required to overcome current limitations in the design of novel therapies to treat neurodegenerative disease

Area of opportunity	Current limitation	Research required
1) Animal and in vitro disease model development.	- Modeling the multifaceted aspect of neurodegenerative disease in vitro. - Addressing the polygenic nature of disease in vitro or in animals. - Translatability of animal models of disease.	- Increased cellular complexity and modeling of cell-cell interactions over time. - Incorporation of vascular elements into multicellular in vitro models. - Generation of polygenic animal or cellular models of disease. - Patient-derived in vitro cell and organoid development. - Address protein isoform and spatiotemporal differences in disease risk and development (e.g., APOE2/3/4, and insoluble fibril versus soluble/oligomeric forms of Aβ). - Epigenetic and ’environmental’ contributions to disease. - Determine sex-based differences. - Determine if models are accurately representing manifestations in human disease.
2) Neurovascular breakdown in disease.	- Clear understanding of the temporal neurovascular events that contribute to neurodegenerative disease.	- Increased understanding of neurovascular coupling mechanisms and pathways. - Detection of the changes in the neurovasculature in vivo over time before and after disease onset.
3) In vivo imaging tools.	- Obtaining single cell resolution in vivo.	- Generation of novel imaging systems that can detect changes at the cellular level, non-invasively in humans and in animal models.
4) Biomarker development.	- Robust, early detection of disease-related biomarkers. - Most of current detection and diagnosis methods were developed using participants of Caucasian/euro-centric origin. - Many patients have comorbid diseases.	- Determination of the key prodromal changes and symptoms for each neurodegenerative disease. - Larger patient cohorts to generate more robust identification of putative biomarkers. - Improved high-throughput molecular systems to detect changes in proteins/biofluids/genes. - Address differences in disease risk stratified by sex, ethnicity, and other diverse populations. - Develop biomarkers for differential diagnosis, recruitment, and keeping in mind cross-disease, co-morbidities for improving clinical trial recruitment (better representing a diverse population). - Differential diagnosis amongst different types of glaucoma, Alzheimer’s, and related dementia, including how to address the common mixed etiology presentation of dementia. - Transcriptomic analysis of cell-type specific changes in models of development and degeneration.

## Data Availability

Data sharing is not applicable to this article as no datasets were generated or analyzed during the current study.

## Abbreviations

Aβ: Amyloid-beta
AD: Alzheimer’s disease
ALS: Amyotrophic lateral sclerosis
APP: Amyloid precursor protein
ATP: Adenosine triphosphate
BBB: Blood-brain barrier
BRB: Blood-retinal barrier
CNS: Central nervous system
CSF: Cerebrospinal fluid
DARC: Detection of apoptosing retinal cells
GWAS: Genome-wide associate studies
HD: Huntington’s disease
HDL: High-density lipoprotein
hiPSCs: Human-derived induced pluripotent stem cells
HSPs: Heat-shock proteins
IOP: Intraocular pressure
IP-TNTs: Inter-pericyte tunneling nanotubes
LHON: Leber’s hereditary optic neuropathy
MRI: Magnetic resonance imaging
MtDNA: Mitochondrial DNA
NSAIDs: Non-steroidal anti-inflammatory drugs
NVU: Neurovascular unit
OCT: Optical coherence tomography
OCT-A: Optical coherence tomography angiography
ONH: Optic nerve head
OPC: Oligodendrocyte precursor cell
PD: Parkinson’s disease
PET: Positron emission topography
PNS: Peripheral nervous system
POAG: Primary open-angle glaucoma
PRGN: Progranulin
PRS: Polygenic risk score
RGC: Retinal ganglion cell
ROS: Reactive oxygen species
SNPs: Single nucleotide polymorphisms
SOD: Superoxide dismutase
TDP-43: TAR DNA-binding protein 43
TREM-2: Triggering Receptor Expressed On Myeloid Cells 2
VCID: Vascular cognitive impairment and dementia
